# Stain-free artificial intelligence-assisted light microscopy for the identification of blood cells in microfluidic flow

**DOI:** 10.3389/fbinf.2025.1628724

**Published:** 2025-08-14

**Authors:** Alexander Hunt, Holger Schulze, Kay Samuel, Robert B. Fisher, Till T. Bachmann

**Affiliations:** ^1^ Centre for Inflammation Research, Institute for Regeneration and Repair, The University of Edinburgh, Edinburgh, United Kingdom; ^2^ Tissues, Cells and Advanced Therapeutics, Scottish National Blood Transfusion Service, NHS National Services Scotland, Jack Copland Centre, Currie, United Kingdom; ^3^ School of Informatics, The University of Edinburgh, Edinburgh, United Kingdom

**Keywords:** artificial neural network, morphological analysis, YOLO v4, blood analysis, haematology

## Abstract

The identification and classification of blood cells are essential for diagnosing and managing various haematological conditions. Haematology analysers typically perform full blood counts but often require follow-up tests such as blood smears. Traditional methods like stained blood smears are laborious and subjective. This study explores the application of artificial neural networks for rapid, automated, and objective classification of major blood cell types from unstained brightfield images. The YOLO v4 object detection architecture was trained on datasets comprising erythrocytes, echinocytes, lymphocytes, monocytes, neutrophils, and platelets imaged using a microfluidic flow system. Binary classification between erythrocytes and echinocytes achieved a network F1 score of 86%. Expanding to four classes (erythrocytes, echinocytes, leukocytes, platelets) yielded a network F1 score of 85%, with some misclassified leukocytes. Further separating leukocytes into lymphocytes, monocytes, and neutrophils, while also increasing the dataset and tweaking model parameters resulted in a network F1 score of 84.1%. Most importantly, the neural network’s performance was comparable to that of flow cytometry and haematology analysers when tested on donor samples. These findings demonstrate the potential of artificial intelligence for high-throughput morphological analysis of unstained blood cells, enabling rapid screening and diagnosis. Integrating this approach with microfluidics could streamline conventional techniques and provide a fast automated full blood count with morphological assessment without the requirement for sample handling. Further refinements by training on abnormal cells could facilitate early disease detection and treatment monitoring.

## Graphical Abstract

**Figure:**
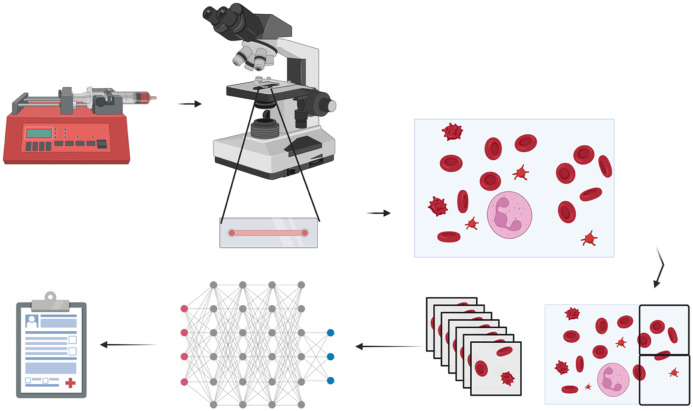
Created with BioRender.com.

## Introduction

The first step to identifying the presence of most pathologies is typically identifying and quantifying blood cells. Full blood counts (FBC) are the most common way to do this. Automated haematology analysers provide population-level data, including total counts, size distributions, and cell type-specific information, revealing valuable clues about underlying conditions (The association of Clinical Biochemistry and Laboratory Medicine, 2023). Utilising technologies like impedance, flow cytometry, and laser diffraction, they are extensively used in clinical practice for swift and precise measurement of blood cell parameters, providing essential results for diagnosis and management of haematological conditions, such as anaemia, leucocytosis, and thrombocytopenia (Chhabra, 2018). However, these techniques are susceptible to interference by factors such as cell agglutination, blood protein concentration and glucose levels. This may cause false measurements of multiple factors of the full blood count, potentially leading to misdiagnosis and or requiring follow-up tests such as a blood smear (Gula et al., 2022).

Over the past few years, extensive research has been devoted to training various neural networks to recognise features in biological samples (Khandekar et al., 2021; Liu et al., 2023; Yang et al., 2020) to infer the presence or absence of specific disease types within specific tissues (Prinzi et al., 2023). Several research teams have investigated training object detectors to identify blood cell types within human blood smears. For example, applying artificial neural networks to the task of detecting the presence of the malaria parasite within fixed and stained erythrocytes in blood smears. The Cascading You Only Look Once (YOLO) approach combines YOLOv2 with a classifier to improve mean average precision by approximately 8% for Plasmodium vivax detection (from 71.34% to 79.22%; Yang et al., 2020). Transformer-based approaches with multiheaded attention mechanisms attained a testing accuracy of 96.41% on original datasets and 99.25% on modified datasets (Islam et al., 2022). AIDMAN, implementing YOLOv5 with an attentional aligner model, demonstrated 98.44% accuracy in clinical validation (Liu et al., 2023). These methodologies offer promising alternatives to traditional microscopy in resource-constrained environments. Others have tried to apply this technique to the detection of circulating blood cancers such as leukaemia from blood smear slides, where a YOLOv4 model was implemented for blast cell detection in acute lymphoblastic leukaemia, achieving a mean average precision of 96.06% for the acute lymphoblastic leukaemia image database for image processing (ALL-IDB1) dataset and 98.7% for the C_NMC_2019 dataset (Khandekar et al., 2021). Blood smears serve as a valuable method for evaluating the morphology of various blood cells. Nevertheless, this process depends on skilled technicians and involves complex, time-consuming laboratory techniques to prepare material (Khandekar et al., 2021; Liu et al., 2023; Yang et al., 2020; Liu et al., 2017).

The published convolutional neural networks designed for hematopathology primarily rely on blood smear images extracted from patient biopsies or online databases (Hu et al., 2022). The accuracy of an artificial neural network model relies on precise training annotations. Challenges arise with peripheral blood film whole slide images due to significant variability in staining techniques, intensity, and colour across laboratories (Fan et al., 2023). To mitigate the potential drawbacks of such variations, the network detailed in this investigation underwent training using stain-free brightfield images. To current knowledge, only one other research team using a microfluidic flow imaging system has focused on immune cell detection, and this relied on using beads binding to CD4 receptors for the identification of target cells (Dixit et al., 2024).

Traditional blood cell classification methods like flow cytometry and immunofluorescence staining require expensive reagents, complex equipment, and skilled operators, limiting their use in resource-constrained settings. These approaches also involve time-consuming sample preparation and staining protocols unsuitable for urgent diagnostics. Our goal was to develop a low-cost, open-source alternative using unstained brightfield images for rapid cell classification. This eliminates costly antibodies and dyes while enabling near-real-time analysis. Unlike previous methods that rely on high-resolution images of fixed, stained cells, our approach uses dynamic flow-based imaging of live cells, requiring a novel workflow that prevents direct comparison to existing datasets.

Using artificial neural networks for high-throughput morphological classification of white blood cells (WBCs) without smearing or staining offers the potential to yield more objective, rapid, cost-effective, and accurate results, eliminating extensive training. The network selected for the task is Yolo-Darknet, coded by AlexeyAB (Bochkovskiy et al., 2020), and all models have been trained using YOLO v4 (Bochkovskiy, 2023). The proposed YOLOv4 architecture has a relatively low inference time of about 1.25 image frames (416*416 pixels) per second. Capitalising on this characteristic, three classification problems were investigated: binary discrimination between erythrocytes and echinocytes, a 4-class blood cell type discrimination (echinocytes, erythrocytes, leukocytes and platelets), and a six-class discrimination (erythrocytes, echinocytes, lymphocytes, monocytes, neutrophils and platelets). Employing a methodology where the camera lens was directed over microfluidic chambers, this system could facilitate real-time identification of WBCs within a concise timeframe, potentially minutes rather than hours, obviating the necessity for extensive blood sampling. This could pave the way to producing results akin to the full blood count and integrating a smear-like morphological analysis in one step without staining or sample handling.

The YOLO family of architectures was particularly suitable for cell classification tasks due to its ability to detect and classify multiple objects within a single pass, making it ideal for analysing densely populated microscopy fields. YOLOv4 was selected as the optimal architecture due to several significant advantages over alternative object detection approaches. YOLOv4 represented the most advanced iteration of the YOLO architecture available, offering substantial improvements in both speed and accuracy (Bochkovskiy et al., 2020). In contrast to earlier implementations, like the Cascading YOLO approach, which needed an additional classifier to detect malaria parasites with an increased precision of 79.22%. The improved feature extraction capabilities of YOLOv4 offered better performance without the need for additional components (Yang et al., 2020). Strong detection of minute morphological variations among blood cell types is made possible by the architecture’s CSPDarknet53 backbone, which is necessary for multi-class discrimination tasks. Instead of requiring the computationally demanding region-by-region analysis that characterises R-CNN approaches, YOLOv4 offers a balance between computational efficiency and detection accuracy when compared to alternative architectures (Alzubaidi et al., 2021). For high-throughput blood cell analysis, where many cells need to be rapidly classified, this efficiency is useful. YOLOv4’s ability to operate effectively on conventional hardware makes it suitable for implementation across various clinical settings, including resource-constrained environments. This combination of speed, accuracy, and implementation feasibility made YOLOv4 the ideal foundation for addressing the key challenges of unstained blood cell classification in microfluidic systems.

Hyperparameter optimisation is necessary for neural network training as it directly impacts model performance (Liao et al., 2022). In deep learning architectures such as YOLOv4, several parameters are critical for effective training (learning rate, momentum and decay). The learning rate sets the speed at which the network adjusts its internal model weights between nodes. Momentum enhances the optimisation process by integrating information from prior weight updates, aiding the model to avoid becoming worse at classifying objects. Finally, weight decay inflicts penalties on large weight values to avoid overfitting, a scenario in which the model performs excellently on training data but fails to classify novel data. These parameters interact in complex ways, requiring systematic fine-tuning. In biological image analysis applications, where subtle morphological differences distinguish between cell types, proper hyperparameter selection becomes particularly important for achieving the discrimination capacity needed for accurate multi-class detection in microscopic blood cell samples.

The present article describes an AI image analysis method for rapidly classifying six major blood cell types: erythrocytes, echinocytes, lymphocytes, monocytes, neutrophils, and platelets. The proposed approach utilises cutting-edge AI techniques to analyse images of blood cells at the microscopic level, enabling the rapid identification of various cell types. Importantly, the accuracy of this AI-driven methodology indicates its promise as a powerful and economical alternative for blood cell classification. This AI methodology is positioned as a promising tool for numerous applications in haematology and clinical diagnostics because of its excellent accuracy and multi-class identification capabilities.

## Materials and methods

A total of 28 healthy volunteer donors from the Centre for Inflammation Research (CIR) Blood Bank were recruited to provide blood for this project. A questionnaire ensured the donor did not use medication or had no early signs of infection (CIR-21-EMREC-041). Each donation was logged, and anonymised records were kept at the CIR Blood Bank. Blood samples were collected by venous phlebotomy and were analysed on the same day with minimal storage (room temperature, 20°C). Each donation consisted of two 5 mL samples of blood collected in EDTA to prevent clotting.

Blood samples were spun at 400 g for 30 min without brake at room temperature. Plasma was collected as a source of platelets and the red cell pellet was resuspended and diluted in PBS pH 7.4 (1:200). Echinocytes were formed by incubating erythrocytes in HEPES buffer pH 8 overnight (37°C).

### Density gradient centrifugation

To achieve a reliable dataset to train the artificial neural network to recognise and distinguish between white cell subpopulations, it was necessary to isolate each subpopulation from whole blood. The white cell subtypes focused on were lymphocytes, monocytes, and neutrophils.

Initially, 5 mL of whole blood was diluted 1:1 with PBS, layered on Ficoll Plaque Plus (GE17-1440-02, Sigma Aldrich, Missouri, United States) and then spun at 450 g for 35 min with both acceleration and brake set to medium. The supernatant was discarded by aspiration, and the interface containing few neutrophils and most monocytes and lymphocytes were washed twice by centrifugation at 350 *g* for 5 min in PBS. Washed cells were resuspended in RPMI medium without glutamine (ThermoFisher, Waltham, United States) supplemented with 10% FBS (ThermoFisher, Waltham, United States) and transferred to a 75 cm^2^ tissue culture flask. After 2 h of incubation at 37°C, the non-adherent lymphocyte supernatant was collected, and the flask was rinsed with PBS. The pooled cell suspension was centrifuged at 350 *g* for 7 min and resuspended in protein-rich PBS solution (PBS pH stabilised at 7.4 with 1% FBS, yielding a suspension enriched for lymphocytes).

TrypLE Express Enzyme solution (ThermoFisher, Waltham, United States) was added to the flask to detach adherent monocytes from the tissue culture plastic. After incubation at 37°C for 10 min, detached cells and PBS flask washes were pooled, cells were centrifuged at 350 *g* for 7 min, and the pellet was resuspended in PBS (buffered at pH 7.4), giving enriched monocytes.

The cell pellet from Ficoll separation, comprising erythrocytes and neutrophils, was washed by centrifugation in PBS before adding red cell lysis solution (Biolegend, San Diego, CA, United States) for 7 min at room temperature. The sample was centrifuged at 350 *g* for 7 min to recover enriched neutrophils. The pellet was washed with PBS and resuspended in RPMI + 10% FBS. Where erythrocyte contamination was still high, by visually checking the colour of the tube, lysis was repeated for 3 min.

### Microscopy observation and image capture

**FIGURE 1:**
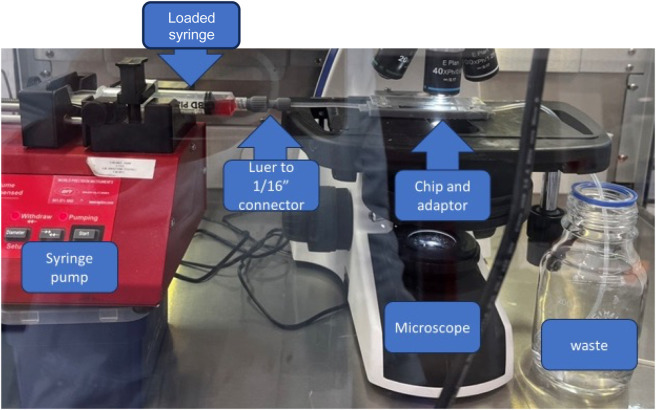
Final assembly of the device prototype. Assembly comprises the syringe pump, a syringe with sample connected to a 3D printed Luer to 1/16 tubing connector, and a microscope with a chip and chip inverter.

**FIGURE 2:**
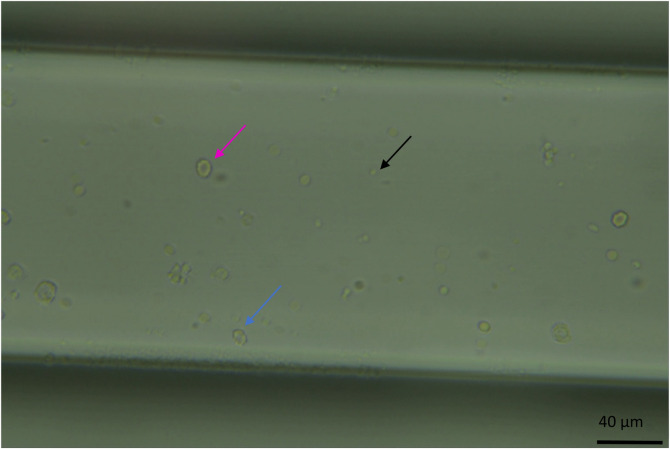
Image of the microfluidic chip channel under the microscope at standard observation settings (x40 zoom, the channel width is 200 μm and 50 µm in depth, whole blood diluted 1:200). Visible here are erythrocytes, a circular biconcave shape (pink arrow). Echinocytes, like erythrocytes, have a biconcave shape but with a more deformed morphology (the cell appears bubbling, blue arrow). And platelets as smaller circular dots (black arrow).

A standard brightfield microscopy procedure was followed to limit variation between observations to obtain the data to train the artificial intelligence-based cell classifier. Initially, data was acquired using a microfluidic chip of 200 µm width, 2 cm length, and 50 µm depth (Chip 156, Microfluidic ChipShop. GmbH, Jena, Germany) observed using an upright light microscope (UltraBio-6 microscope, GT Vision, Wickhambrook, United Kingdom) equipped with a Basler asA3088-57uc camera (Basler AG, Ahrensburgh, DE, Figure 1). Finally, a chip holder was designed to interface with the microscope and keep the microfluidic connections available once placed under the microscope. This enabled channel switching during the experiment and observation of the entire channel length from entry to exit, with the additional benefit of keeping the focus distance constant. Once the chip was connected to the light microscope, the microscope was set to the 40x zoom lens and adjusted so the chip channel was in focus. The pylon software (version 7.2.1, Basler AG, Ahrensburgh, DE) was set up to acquire a series of still image files by saving 100 frames per second, allowing the circulation of the cells in the field of view, capturing a new set of cells randomly scattered throughout the observable area (Figure 2).

To collect erythrocyte/echinocyte data, whole human blood samples were diluted 1:200 in PBS (pH 7.4) before loading 20 µL of the diluted blood sample into one channel of the chip. Without dilution, the field of view was too crowded to distinguish the features of individual cells (Supplementary Figure S2).

For training purposes, digital images were acquired for each isolated blood cell sub-population and loaded into the chip separately. Cell numbers/concentrations varied between fractions depending on the sample, sometimes requiring the collection of more data points. For this, three independent, 5 mL donations were collected from 14 donors. For each donation, a set of 150 full-frame images were extracted from recordings to populate the training and validation dataset. An additional 15 full-frame images per donation were extracted and isolated separately for testing. Each frame is split into 414 × 416 pixel tiles with, on average, 4 cells (±3 cells) per tile (with about 60 tiles generated from a full-frame image).

### Randomizing sample composition

To demonstrate that the device could identify atypical sample composition, the different isolated components, red cell concentrate, fresh plasma, and leukocyte concentrates, were reassembled at different concentrations of erythrocytes than typical samples (from 1:1,000 to 1:1 in increments of 50). Twenty samples were split into pairs; one of each pair was analysed by the device, and the other was labelled and analysed by flow cytometry. Ratios of cell types were calculated from the total sample cell count and plotted against each other. Multiple t-test analysis was performed on the ratios to determine if they were statistically similar to one another.

### Flow cytometry

#### Sample validation

Cell suspensions were analysed by multiparametric flow cytometry on a BD Fortessa instrument (BD Biosciences). White cell suspensions were stained separately from red cells and platelet suspensions to quantify cell counts, size and population diversity.

Anti-human CD45 (BV421), CD14 (PE) and CD16 (APC) antibodies were used to stain the white cell suspensions. Anti-human CD71 (FITC) and CD235a (PE) were used to stain platelets and red cell suspensions (BioLegend, San Diego, CA, United States).

To prepare a sample for analysis from full blood, 100 μL of blood was set aside from the main experiment. From the sample, 10 μL were diluted into 290 μL of FACS PBS (pH 7.2, with 5% FBS and 0.1% sodium azide), and 1 μL of CD71 and 1uL of CD235a were added to stain the cells. After 20 min of incubation at 4C, cells were washed with PBS and centrifuged at 350 *g* × 7 min before the pellet was resuspended in 200 µL PBS+0.5% FCS. The sample was stored at 2°C–6°C, fixed in formalin, overnight prior to data acquisition.

To analyse leukocyte populations, 1 μL each of CD14, CD16, and CD45 was added to the remaining 90 μL of the sample after incubation for 20 min at 2°C–6°C 210 μL PBS (pH 7.2) was added before centrifugation at 350 *g* for 7 min 500 μL of fix and lyse solution (BD) was added to the pellet. After incubation for 5 min at room temperature, samples were spun at 350 g for 7 min to remove the supernatant and lysed cells. The pellet was resuspended in 300 μL of FACS PBS and stored at 2°C–6°C overnight prior to data acquisition.

#### Data acquisition

Unstained and single-colour compensation controls were used to calculate compensation matrices for data acquisition. Spectral overlap was automatically calculated and compensated using BD CompBead particles. At least 100,000 events were recorded for each sample using the BD FACSDiva software. The final analysis was performed using FlowJo v10 software (BD), and a gating strategy was applied for analysis (supplementary data).

### Benchmark tests: flow cytometry and haematology analysers

Flow cytometry which can quickly process a whole sample and is the gold standard technology for cell counting and phenotypic analysis (Power et al., 2021) was used to verify test results from neural network classifier testing. Once the sample had run through the artificial neural network training system, a sample of the initial donation and of each isolated leukocyte sub-population was stained and processed the same day as fixing, using a BD Fortessa-SORP flow cytometer. Both BD FACSDIVA 8.0.1 and BD FlowJo software were used for data acquisition and analysis.

Whole blood counts for undiluted blood were acquired using a Beckman coulter DxH520 analyser, quality controlled using standards provided by the manufacturer. For flow cytometry analysis of samples, data was acquired using the same parameter settings for each sample. To accurately assess red cell and platelet frequency, diluted whole blood was used, whereas leukocyte-sub-populations were assessed on erythrocyte-depleted samples. Data was analysed in Flowjo; electronic gates were applied to identify the percentage of sub-populations of interest. The data from the haematology analyser, flow cytometry and prototype were compared in a three-way ANOVA using GraphPad Prism 10.2.1 for statistical analysis.

#### Data pre-processing and augmentation

The Basler Pylon software suite captures video files that are converted to still images in JPEG format to ensure compatibility with the Python neural network software. Isolated subsets of cells were annotated as a collection before moving to the next subset to minimise mislabelling. Images were labelled using LabelIMG by Tzutalin, and annotations were exported in YOLO format (in a text file; Tzutalin, 2020). As mentioned previously, image sets from isolated cells were annotated per the isolated type to reduce error. Each subtype would begin with 50 full-frame annotated images prior to cropping. Annotated images were then cropped into subdivisions of 416 × 416 pixels, and annotations were automatically mapped onto the new images using the new cropped image coordinates using custom Python 3 scripting and OpenCV libraries (‘opencv/opencv’, 2024, Supplementary Figure S1). Annotations are automatically removed if the cell is within 5 pixels of the side of the images to ensure the neural networks train on fully visible cells.

The training subset of the dataset of cropped images and annotations is augmented by applying transformations to the original images, including rotating the images (90, 180 and 270°), flipping the images (along both the horizontal and vertical mid axes), changing contrast (±5%), brightness (±5%), saturation, hue, sharpness, and focus (±5%) to create a varied dataset. Optional steps can be applied, such as removing or remapping one or more classes from the original dataset to generate a dataset for specific purposes (such as detecting and discriminating between two cell lines, troubleshooting, and increasing the number of examples of a particular class of objects). This is done to increase the size of the training set and increase variability within the sample that the classifier can train on.

#### Artificial neural network training and testing

The dataset described above was used to train multiple neural network models for cell detection and classification, including the primary YOLOv4 architecture, various configurations of YOLOv4 with different hyperparameters, and for comparison purposes, YOLO versions 5 through 7. The selected backbone for training and inferring is the Yolo-Darknet coded by AlexeyAB, and all models have been trained on the YOLO v4 (Bochkovskiy, Wang and Liao, 2020; Bochkovskiy, Alexey, no date). Each model was trained for ten thousand epochs (the number of times each image is passed to the CNN). The system supports batch processing with adjustable subdivision parameters and implements advanced training techniques, including learning rate scheduling, burn-in periods, and mosaic data augmentation. Each model was trained with a progressively larger dataset: the binary classification network (echinocytes and erythrocytes) used over 9,000 image tiles, the four-class network (echinocytes, erythrocytes, leukocytes, and platelets) used around tile 12,000 images, and the six-class network (echinocytes, erythrocytes, lymphocytes, monocytes, neutrophils, and platelets) used the largest dataset of over 197,000 images.

For testing, a subset of the training dataset is set aside (roughly 10% of the total number of images). Those images are then used to independently evaluate performance by comparing the inference of the trained model to the human-defined labels. The comparison between predicted and ground truth bounding boxes was performed using IoU. An IoU threshold of 75% was used to determine if a detection was considered correct. Using functions from Matplotlib (a plotting library for creating visualisations and graphs), Seaborn (a statistical data visualisation library built on Matplotlib that enhances the aesthetics of plots), and Pandas (a data manipulation library for structured data analysis) libraries in Python3 allows for quick analysis of the similarities and discrepancies of the produced output. This generates comparable metrics for each developed model, including visualisations of bounding box areas, neural network test scores (precision, recall, f1 score), and class distributions. This was necessary to compare the predicted and labelled bounding boxes and calculate the accuracy (the proportion of samples that were correctly identified to the entire set), precision (the portion of correctly identified instances over the total number of instances), recall (synonymous to a diagnostics test’ sensitivity) and F1 score (combines both precision and recall into one value, providing a balanced measure of a model’s performance in classifying entities; Hicks et al., 2022).

To evaluate the network’s capacity to detect rare cell types within a predominantly different cell population, a series of dilution experiments using mixed image datasets were performed. Erythrocyte cells were systematically diluted in echinocyte populations at ratios of 1:10, 1:100, and 1:1,000 (erythrocyte). For each dilution, a composite dataset by combining images of both cell types at their respective proportions was created. The datasets were prepared by randomly sampling from the dataset of erythrocytes and echinocyte annotated images. The detections were obtained from pre-trained YOLOv4 algorithms and followed the testing pipeline outlined above.

The classifiers were trained and tested using five-fold cross-validation, an evaluation technique where the dataset is divided into five equal parts. Combining all the original image data within one folder and creating five subsets of the data. Those five subsets were then randomly redistributed five times into three sets: one each for training, validation, and testing. This allowed statistical analysis with a standard deviation of the acquired data, providing a more reliable assessment of the model’s performance than a single train-test split. The original image data is split into independent cropped images for each training regimen, each with an independently detected single cell. All models were trained with stratified 5-fold cross-validation where all data was pooled and then split into training (70%), validation (20%) and testing (10%). The stratified approach ensures that each fold maintains the same proportion of samples from each class as the complete dataset, which is crucial for imbalanced datasets like this one with varying numbers of each cell type. Then, each model was trained and tested five times (once per shuffling combination).

True positive, false positive, and false negative were calculated per class and overall to assess the detection power of the trained models. In the context of object detection with bounding boxes. True positives are detections where the model correctly identifies both the class of the object and its location. This is determined when the IoU between the predicted bounding box and the ground truth bounding box exceeds the threshold (75% in this case) and the predicted class matches the ground truth class. False positives occur in two scenarios: when the model predicts a bounding box where no object exists or when it correctly identifies the presence of an object but assigns the wrong class label to it, or when the IoU between the predicted bounding box and the ground truth is below the threshold. False negatives occur when the model fails to detect an object that is present in the ground truth annotations. This happens when no predicted bounding box has sufficient overlap (IoU > threshold) with a ground truth bounding box.

The system also includes functions to generate comprehensive reports with detailed metrics, export error images for qualitative assessment, and conduct inference on new samples with confidence scoring. This end-to-end pipeline provides a robust framework for blood cell detection and classification based on morphological features detectable in microscopy images. The full code repository is available at the following repository: https://github.com/alex1075/machine-code.git.

## Results

### Two class discrimination: echinocytes and erythrocytes

Testing the suitability of YOLO v4 for discriminating between two unstained cell populations began with discrimination between echinocytes and erythrocytes. Table 1A summarises the results of the YOLOv4 model’s predictions for echinocytes and erythrocytes from one-fold of a 5-fold cross-validation experiment. For echinocytes, the model achieved a precision of 93.8% ± 0.9%. The recall for echinocytes was 78.1% ± 1.2%, indicating that the model correctly identified approximately 78% of all actual echinocytes, with the remaining either misclassified or undetected. The F1 score, which balances precision and recall, was 85.3% ± 0.8% (Table 1B A few examples of this are visible in Supplementary Figure S3; most of the cells were correctly identified. However, one instance of echinocytes was identified as erythrocyte. For erythrocytes, the model demonstrated a precision of 84.8% ± 1.0%, recall of 88.9% ± 2.5%, and an F1 score of 86.7% ± 1.0%. Notably, the model achieved higher recall for erythrocytes than for echinocytes, suggesting it was more effective at identifying erythrocytes when they were present. However, the precision for echinocytes was higher, indicating fewer false positives when classifying echinocytes. Overall, the network achieved a macro-average precision of 89.3% ± 0.8%, recall of 83.5% ± 0.8%, and F1 score of 86.0% ± 0.6% (Table 1B.)

**TABLE 1:** A. Confusion matrix of the YOLOv4 model trained to discriminate between Echinocytes and Erythrocytes B. Detailed breakdown of the Precision, Recall and F1 score per blood cell subtype of the 5-fold cross validation of the binary network. Values are in percentage.

A				
Ground truth	Echinocyte	408	66	56
	Erythrocyte	29	349	9
		Echinocyte	Erythrocyte	Non detected
		Prediction		

B			
Cell type	Precision	Recall	F1 score
Echinocytes	93.8 ± 0.9	78.1 ± 1.2	85.3 ± 0.8
Erythrocytes	84.8 ± 1.0	88.9 ± 2.5	86.7 ± 1.0
Network	89.3 ± 0.8	83.5 ± 0.8	86.0 ± 0.6

The confusion matrix reveals specific error patterns: of the echinocytes, 77.0% were correctly classified, 12.5% were misclassified as erythrocytes, and 10.5% went undetected. For erythrocytes, 90.2% were correctly classified, 7.5% were incorrectly classified as echinocytes, and only 2.3% were not detected. These results suggest that while the model is effective at discriminating between these cell types, it faces greater challenges in detecting and correctly classifying echinocytes compared to erythrocytes.

Looking at the macro average of precision and recall for the model, it is possible to see that the network achieves solid performance with 89.3% ± 0.8% precision and 83.5% ± 0.8% recall across both cell types. The overall F1 score of 86.0% ± 0.6% indicates a good balance between precision and recall. These results suggest the model is reliable when classifying echinocytes and erythrocytes without staining, though it shows slightly better performance with erythrocytes. The network’s demonstrated ability to discriminate effectively between these two morphologically distinct red blood cell types provided a strong foundation for subsequent experiments with additional cell types.

After initial training, network optimisation was performed by modulating training hyperparameters to tune the neural network to the task of blood cell classification. For this, momentum was modulated between 0.93 and 0.97 in increments of 0.01. Learning rate was modulated between 0.00001 and 0.1 in increments of 10. And Decay was modulated between 0.005 and 0.00005 in increments of 10 (detail of the modulation in Supplementary Table S1). Once each network has been trained and tested, the results can extract the best-performing parameters for the application of blood cell detection which are from network beta-A-3 with a learning rate of 0.93, momentum of 0.0001 and decay of 0.00005 (Supplementary Figure S4). By comparing the resulting metrics from each trained network from Supplementary Table S2, the best performing parameters were selected and found to be from the beta-A-3, Supplementary Figure S4. This set of parameters resulted in a precision of 91.6%, a recall of 90.4%, and an F1 score of 91%. These parameters were subsequently applied to all network training.

Table 2A summarises the results of the classification model’s predictions for echinocytes and erythrocytes, which are two phenotypes of the same cell type. For echinocytes, the model achieved a precision of 90%. The recall for echinocytes was 82.6%, signifying that the model correctly identified approximately 83% of all actual echinocytes. The F1 score, which balances precision and recall, was calculated at 86.2% (Table 2B). Similarly, for erythrocytes, the model demonstrated a precision of 93.9%, recall of 95.9%, and an F1 score of 95.0%. The confusion matrix reveals that out of 98 echinocytes, 81 were correctly classified, 15 were misclassified as erythrocytes, and 2 remained undetected. For erythrocytes, 232 out of 242 were correctly classified, with 9 misclassified as echinocytes and 1 undetected. These metrics collectively indicate the model’s effectiveness in discriminating between echinocytes and erythrocytes, with an overall network performance of 91.6% precision, 89.2% recall, and 90.5% F1 score (Table 1B).

**TABLE 2:** Binary YOLOv4 model results. A. Confusion matrix of a one-fold of the 5-fold cross validation of the optimised YOLOv4 model (version beta-A-3) trained to discriminate between Echinocytes and Erythrocytes B. A detailed breakdown of the Precision, Recall and F1 score per blood cell subtype. Values are average percentage.

A				
Ground truth	Echinocyte	81	15	2
	Erythrocyte	9	232	1
		Echinocyte	Erythrocyte	Non detected
		Prediction		

B			
Cell type	Precision	Recall	F1 score
Echinocytes	90	82.6	86.2
Erythrocytes	93.9	95.9	95.0
Network	91.6	89.2	90.5

### Four class discrimination: echinocytes, erythrocytes, leukocytes and platelets

Leukocyte and platelet image data were added to the 2-component dataset. This was done using the acquisition of images from platelets and leukocytes isolated into individual populations (lymphocytes, monocytes, and neutrophils) from one another and labelled as such. The class IDs were then remapped to combine the data for all 3 cell types into a single class leukocyte class before the network training. The 4-component model demonstrated effective discrimination between different blood cell types as shown in Table 3A. For echinocytes, the model achieved a precision of 93.3% ± 3.1% and recall of 78.9% ± 6.8%, with an F1 score of 85.4% ± 5.1%. The confusion matrix reveals that out of 542 echinocytes, 429 were correctly classified, while 43 were misclassified as erythrocytes, 6 as leukocytes, 6 as platelets, and 58 remained undetected. Erythrocyte detection showed a precision of 83.7% ± 6.7% and recall of 79.6% ± 10.6%, with 261 correctly identified out of 311 total erythrocytes.

**TABLE 3:** Four class YOLOv4 testing results. A. Confusion matrix of one-fold of a five-fold validation trained YOLO-V4 model trained to discriminate between Echinocytes, Erythrocytes, Platelets and White Blood cells (leukocytes). B. Confusion matrix of the same model trained with Echinocyte detections, remapped as Red Cells C. Detailed breakdown of the Precision, Recall and F1 score per blood cell subtype. D. Detailed breakdown of the Precision, Recall and F1 score per blood cell subtype after remapping echinocytes and erythrocytes as red cells. For both breakdowns, values are average percentage with standard deviation.

A						
Ground truth	Echinocyte	429	43	6	6	58
	Erythrocyte	24	261	1	7	18
	Leukocyte	1	2	116	0	26
	Platelet	8	5	2	148	11
		Echinocyte	Erythrocyte	Leukocyte	Platelet	Non detected
		Prediction				

B					
Ground truth	Red cells	757	7	13	76
	Leukocyte	3	116	0	26
	Platelet	13	2	148	11
		Red cells	Leukocyte	Platelet	Non detected
		Prediction			

C			
Cell type	Precision	Recall	F1 score
Echinocytes	93.3 ± 3.1	78.9 ± 6.8	85.4 ± 5.1
Erythrocytes	83.7 ± 6.7	79.6 ± 10.6	81.0 ± 5.4
Platelets	88.8 ± 3.2	78.6 ± 7.4	83.3 ± 5.1
Leukocytes	89.9 ± 5.2	85.2 ± 5.2	87.5 ± 4.7
Network	89.1 ± 3.5	80.3 ± 1.2	84.2 ± 1.9

D			
Cell type	Precision	Recall	F1 score
Red cells	98.1 ± 0.6	88.4 ± 1.3	93.0 ± 0.6
Platelets	88.8 ± 3.2	78.6 ± 7.4	83.3 ± 5.1
Leukocytes	90.0 ± 4.5	85.6 ± 3.8	87.6 ± 4.1
Network	92.6 ± 2.9	84.2 ± 1.6	88.1 ± 1.8

Platelet detection demonstrated good performance with a precision of 88.8% ± 3.2% and recall of 78.6% ± 7.4%, correctly identifying 148 of 174 platelets. Leukocytes were detected with a precision of 89.9% ± 5.2% and recall of 85.2% ± 5.2%. Overall, the network achieved macro-average metrics of 89.1% ± 3.5% precision, 80.3% ± 1.2% recall, and an F1 score of 84.2% ± 1.9%.When echinocytes and erythrocytes were combined into a single red cell class, Tables 3B,D, the model’s performance improved significantly. The red cell class demonstrated enhanced precision (98.1% ± 0.6%) and recall (88.4% ± 1.3%), yielding a strong F1 score of 93.0% ± 0.6%. The confusion matrix shows that 757 red cells were correctly identified out of 853 total, with only 7 misclassified as leukocytes, 13 as platelets, and 76 undetected. Leukocyte and platelet detection remained consistent with the 4-class model, with platelets showing 88.8% ± 3.2% precision and 78.6% ± 7.4% recall, and leukocytes demonstrating 90.0% ± 4.5% precision and 85.6% ± 3.8% recall.

The combined 3-class model (red cells, leukocytes, platelets) achieved improved overall network performance with a macro-average precision of 92.6% ± 2.9%, recall of 84.2% ± 1.6%, and F1 score of 88.1% ± 1.8%. This improvement suggests that the model struggles more with distinguishing between the two similar cell phenotypes (echinocytes and erythrocytes) than with differentiating between the more morphologically distinct cell types (red cells, leukocytes, and platelets).

### Six class discrimination: addition of isolated lymphocytes, monocytes, and neutrophils expanding leukocyte class

As opposed to previously where the leukocytes were combined into one single network class, here the leukocytes were labelled per their subtype, lymphocyte, monocyte and neutrophil. There were over 197,000 images comprising: 53,100 echinocytes, 33,200 erythrocytes, 7,100 lymphocytes, 3,300 monocytes, 4,100 neutrophils and 9,600 platelets. The remaining images were a combination of images of the channel background, debris and other non-labelled anomalies.

**FIGURE 3:**
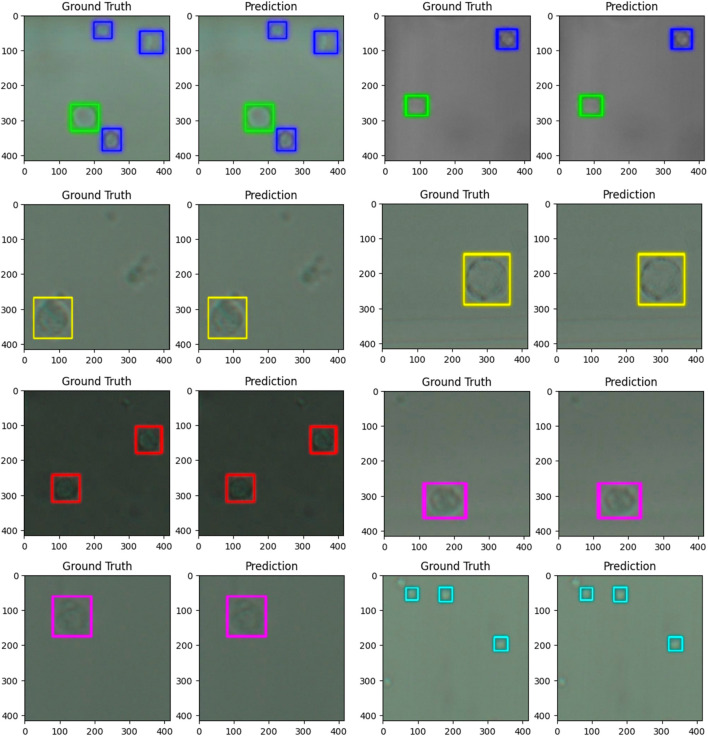
images comparing the ground truth (human label) to the predictions (computer label) for six populations of cells from whole blood. The predictions were generated by a trained model based on the YOLO v4 artificial neural network architecture. The dimensions of the images are 416 × 416 pixels or 57.37 × 57.37 µm. The cells were imaged at 40x zoom. Cells labelled in cyan are platelets, and cells labelled in dark blue are echinocytes, green–erythrocytes, red–lymphocytes, yellow–neutrophils, and pink–monocytes.

As can be seen in Table 4B, the six-class model demonstrated varying performance across cell types. The highest F1 scores were achieved by Platelets (89.0% ± 1.1%) and Neutrophils (79.3% ± 0.7%), while Lymphocytes showed the lowest F1 score (62.1% ± 1.0%). Echinocytes and Erythrocytes achieved moderate F1 scores of 76.3% ± 0.4% and 71.1% ± 0.5%, respectively, despite having the largest representation in the dataset. The confusion matrix in Table 4A illustrates specific misclassification patterns, particularly between Echinocytes and Erythrocytes, with 5,235 Echinocytes misclassified as Erythrocytes and 1,457 Erythrocytes misclassified as Echinocytes. Among leukocytes, Monocytes achieved a moderate F1 score of 67.8% ± 1.5%, with notable confusion occurring between leukocyte subtypes (one of the few errors of the network are outlined in Supplementary Figure S6; Figure 3). These performance metrics provide context for the subsequent improvements observed in the five-class model, where combining Echinocytes and Erythrocytes into a single Red Cells category significantly enhanced the model’s discriminative capability.

**TABLE 4:** Six class YOLOv4 testing results. A. Confusion matrix of one-fold of a five-fold YOLO-V4 model trained to discriminate between Echinocytes, Erythrocytes, Lymphocytes, Monocytes, Neutrophils and Platelets. The model was tested separately from the training and validation dataset. Count represents individual number of cells within the training set of the model. B. Breakdown of A’s model’s performance after training and testing using five-fold validation. Represented is the average with the standard deviation. Values are average percentage with standard deviation. C. Confusion matrix of one-fold of a five-fold retrained model from A. YOLO-V4 model now trained to discriminate between red cells, Lymphocytes, Monocytes, Neutrophils and Platelets. The model was tested separately from the training and validation dataset. Count represents individual number of cells within the training set of the model. D. Breakdown of the C’s model performance after training and testing using five-fold validation. Represented is the average with the standard deviation. Values are average percentage with standard deviation.

A								
Ground truth	Echinocyte	14,174	5235	929	119	45	488	0
	Erythrocyte	1457	10,278	837	146	60	142	0
	Lymphocyte	46	202	1955	77	62	7	0
	Monocyte	31	71	94	954	69	9	0
	Neutrophil	6	23	133	246	1314	0	0
	Platelet	154	129	6	0	0	4609	57
		Echinocyte	Erythrocyte	Lymphocyte	Monocyte	Neutrophil	Platelet	Non detected
		Prediction						

B			
Cell type	Precision	Recall	F1 score
Echinocytes	89.1 ± 0.2	66.7 ± 0.5	76.3 ± 0.4
Erythrocytes	64.5 ± 0.5	79.2 ± 0.5	71.1 ± 0.5
Lymphocytes	50.0 ± 1.1	82.4 ± 0.9	62.1 ± 1.0
Monocytes	61.1 ± 1.1	76.3 ± 2.1	67.8 ± 1.5
Neutrophils	84.5 ± 1.3	74.7 ± 1.0	79.3 ± 0.7
Platelets	84.3 ± 2.5	94.3 ± 0.9	89.0 ± 1.1
Network	72.2 ± 0.7	78.9 ± 0.5	74.3 ± 0.6

C							
Ground truth	Red Cells	30,784	1665	222	86	984	0
	Lymphocyte	271	2021	73	49	6	0
	Monocyte	121	79	947	75	6	0
	Neutrophil	28	160	280	1319	0	0
	Platelet	210	1	0	0	4696	51
		Red Cells	Lymphocyte	Monocyte	Neutrophil	Platelet	Non detected
		Prediction					

D			
Cell type	Precision	Recall	F1 score
Red Cells	97.9 ± 0.1	91.3 ± 0.2	94.6 ± 0.1
Lymphocytes	50.4 ± 1.0	82.9 ± 1.0	62.7 ± 0.9
Monocytes	61.7 ± 0.6	77.2 ± 0.6	68.7 ± 0.5
Neutrophils	85.0 ± 1.4	75.0 ± 1.1	79.4 ± 0.6
Platelets	84.5 ± 2.2	94.2 ± 0.9	79.4 ± 0.6
Network	75.9 ± 0.4	84.0 ± 0.3	78.9 ± 0.3

As shown in Table 4A, the analysis revealed important performance differences between the six-class and five-class YOLOv4 models. In the six-class model (Tables 4A,B), where Echinocytes and Erythrocytes were classified separately, the network achieved an overall F1 score of 74.3% ± 0.6%, with precision and recall values of 72.2% ± 0.7% and 78.9% ± 0.5%, respectively. When these two cell types were reclassified as a single Red Cells category in the five-class model (Tables 4C,D), the network performance improved substantially. The overall F1 score increased to 78.9% ± 0.3%, with precision rising to 75.9% ± 0.4% and recall to 84.0% ± 0.3%. This improvement was particularly pronounced in the Red Cells category, which achieved an F1 score of 94.6% ± 0.1%, significantly higher than the separate scores for Echinocytes (76.3% ± 0.4%) and Erythrocytes (71.1% ± 0.5%) in the six-class model. The performance for other leukocyte classes remained relatively stable between the two models, with slight improvements in Lymphocyte and monocyte detection. These results suggest that combining morphologically similar cell types enhances the model’s discriminative capability and overall performance.

Table 5 presents the performance metrics of the three-class YOLOv4 model retrained from the 6-class network from Table 4, where leukocyte subtypes (lymphocytes, monocytes, and neutrophils) were consolidated into a single Leukocyte class, and erythrocytes subtypes (erythrocytes and echinocytes) were also consolidated to a single Red Cell class. This simplified model demonstrated positive overall performance with a network precision of 84.1% ± 0.9%, recall of 92.4% ± 0.2%, and F1 score of 87.6% ± 0.5%. Red cells were detected with high accuracy, achieving a precision of 97.9% ± 0.1%, recall of 91.2% ± 0.6%, and F1 score of 94.5% ± 0.3%. The consolidated Leukocyte class showed substantial improvement compared to individual leukocyte subtypes in previous models, with a precision of 70.3% ± 1.0%, recall of 91.9% ± 0.5%, and F1 score of 80.0% ± 0.7%. Platelets maintained consistent performance with 84.0% ± 2.8% precision, 94.1% ± 1.0% recall, and 88.8% ± 1.1% F1 score. The confusion matrix reveals that most misclassifications occurred between red cells and leukocytes, with 2,074 red cells incorrectly identified as leukocytes and 448 leukocytes incorrectly identified as red cells. These results demonstrate that consolidating morphologically similar cell types significantly enhances the model’s discriminative capability and overall performance.

**TABLE 5:** A. Confusion matrix of one-fold of a five-fold retrained model from Table 4A YOLO-V4 model re-trained to discriminate between red cells, Leukocytes and Platelets. The model was tested separately from the training and validation dataset. The count represents the individual number of cells within the training set of the model. B. Breakdown of Table 4B’s model performance after re-training and testing using five-fold validation. Represented is the average with the standard deviation. Values are average percentages with standard deviations.

A					
Ground truth	Red cells	30,864	2074	839	2
	Leukocyte	448	4906	19	0
	Platelet	250	1	4737	73
		Red cells	Leukocyte	Platelet	Non detected
		Prediction			

B			
Cell type	Precision	Recall	F1 score
Red cells	97.9 ± 0.1	91.2 ± 0.6	94.5 ± 0.3
Leukocytes	70.3 ± 1.0	91.9 ± 0.5	80.0 ± 0.7
Platelets	84.0 ± 2.8	94.1 ± 1.0	88.8 ± 1.1
Network	84.1 ± 0.9	92.4 ± 0.2	87.6 ± 0.5

#### Visualization of neural network output

**FIGURE 4:**
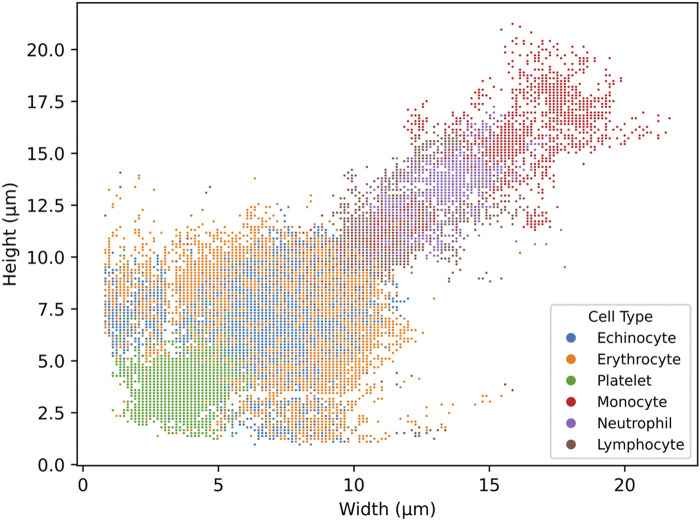
scatterplot of the distribution of cell width and height in µm from the detections generated by the six-class trained YOLO v4 model. The model was trained on six cell subtypes of blood: echinocytes, erythrocytes, platelets, monocytes, neutrophils, and lymphocytes. The scatterplot was generated from a full blood sample.

The neural network’s detection output for whole blood samples can be visualised by plotting the approximate height and width of each detected cell (with a 2 µm margin of error), as illustrated in Figure 4 Interestingly, this representation reveals that the detected cell populations correspond well with their expected size ranges *in vivo*. The scatterplot displays platelets at the lower end, red blood cells in the mid-range (with some variation due to their potential rotation relative to the camera), and a heterogeneous population of white blood cells at the upper end, mostly ranging from 12 to 25 µm in both width and height. While this correlation between the neural network’s output and actual cell sizes is significant, it is essential to emphasise that the neural network’s internal decision-making process remains opaque. The features or characteristics prioritised by the model during classification cannot be definitively determined, and it would be premature to conclude that cell size played a significant role in the detection process. This graph serves to validate the accuracy of the detection rather than to illustrate the method employed by the neural network.

### Randomized blood samples

**FIGURE 5:**
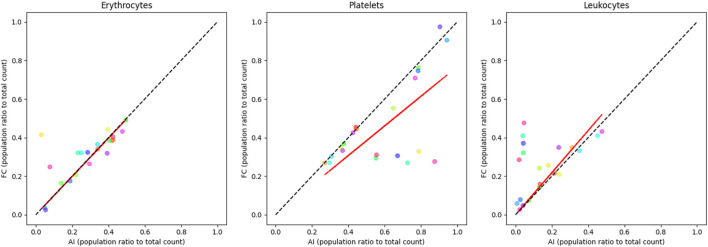
correlation plots of randomised blood cell concentration samples comparing labelled flow cytometry and a custom-trained three-class YOLO v4 object detector (Table 5) major sub-population ratio (trained to recognise echinocytes, erythrocytes, lymphocytes, monocytes, neutrophils, and platelets). Erythrocyte ratios are mostly consistent along the correlation line (R^2^ = 0.5649). Platelets are overrepresented in the AI device (R^2^ = 0.2252), while leukocytes tend to be more detected by flow (R^2^ = 0.3969). Each colour represents a single sample pair. The red line represents the observed trend line, while the dotted black line represents perfect correlation.

As apparent in Figure 5, the comparison of major blood subpopulations between the Table 5 trained three-class network and flow cytometry shows a partial correlation between the two techniques when analysing randomised blood cell concentrations. The statistical correlation test reveals significant relationships between the two techniques, with p-values ranging from 0.0000865 to 0.0297 (Supplementary Table S2). The coefficient of determination values further quantify these relationships, with erythrocytes showing a moderate correlation (R^2^ = 0.5649), leukocytes showing a weaker correlation (R^2^ = 0.3969), and platelets showing the weakest correlation (R^2^ = 0.2252). The observed trend lines for platelets and leukocytes deviate from perfect correlation.

### Comparison to different technologies

**FIGURE 6:**
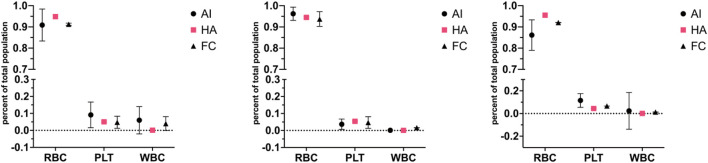
Correlation plots for red, white and platelet cell populations across different cytometry techniques for three different blood donors (prototype device–AI, haematology analyser–HA, flow cytometry–FC). Three donations from three donors (each donation split three ways, one graph per donor), not used for artificial neural network training from Table 5, were run through the microscopy setup, flow cytometry and an NHS lab haemato-analyser. The figure represents the total cell population ratios over total cell counts; throughout the cell types, the ratios remain within the range of one another. The standard deviation is represented in error bars. Statistical analysis of each donor set reveals no statistical difference between techniques (two-way ANOVA, donor 1: p = 0.6063, donor 2: p > 0.9999, donor 3: p > 0.9999). The artificial neural network stands for an artificial neural network device; FC is for flow cytometry, and HA is for haemato-analyser.

To compare the proposed AI method to already established standard methods in haematology, three blood donations were collected from three donors not used for the training of the neural network (used network from Table 5). This ensured that the detection of the network was possible on any blood sample without needing to train on blood from every single individual. Full blood was imaged and analysed by the network using the prototype device, paired samples were also analysed by means of fluorescence-stained flow cytometry and a United Kingdom NHS lab autoanalyzer (Figure 6). As observable, the figure represents the total cell population ratios over total cell counts; throughout the cell types, the ratios remain within the range of one another. Statistical analysis of each donor set reveals no statistical difference between the different techniques where most of the variation between results is caused by the cell types and interaction of cell type and technique (two-way ANOVA, donor 1: p = 0.6063, donor 2: p > 0.9999, donor 3: p > 0.9999). Thus, revealing no difference between the device, flow cytometry analysis and the gold standard autoanalyzer.

### Comparing YOLO version 4 to newer versions of YOLO networks

Figure ƒ compares the different YOLO versions, YOLOv4 and YOLOv7 inclusive, all trained on the modified 3-class network (red cells, leukocytes, and platelets). Since the beginning of the project, newer versions of YOLO have been released. Comparing the newer versions of YOLO to the optimised YOLOv4 running the networks detecting unstained blood cells, it is evident that YOLOv4 is the better performing so far.

## Discussion

### Optimizing YOLO v4 for accurate unstained blood cell binary detection and classification

Currently, no other publication has reported discrimination between entirely unstained live blood cell subpopulations. We, therefore, initiated the development of an artificial neural network for simple binary classification, distinguishing between erythrocytes and echinocytes. The YOLO v4 network was trained to discriminate between the two red cell sub-populations, achieving good overall accuracy (89.3% ± 0.8% precision, 83.5% ± 0.8% recall, and 86.0% ± 0.6% F1 score, Table 2). However, some erythrocytes were misclassified as echinocytes, with the confusion matrix revealing that out of 387 erythrocytes, 29 (7.5%) were incorrectly classified as echinocytes, while 66 (12.5%) of 530 echinocytes were misclassified as erythrocytes. This may be due to potential issues in treatment during the imaging, such as cells reacting differently to treatment in HEPES buffer or cell age at the start of the treatment affecting their morphology (cell lysis) (Melzak et al., 2021). Alternatively, some healthy, untreated red cells may have undergone echinocyte formation during sample collection.

Performing this test was significant as it demonstrated the ability to distinguish between two types of erythrocytes. Indeed, binary artificial neural networks have potential applications, such as detecting cells infected by the *plasmodium* parasite, which has been demonstrated in stained blood smears using modified YOLOv2 and modified YOLOv3 and YOLOv4 binary classifiers. Previous research teams achieved an average precision of 79.22% with stained blood smear data when comparing circulating erythrocytes and erythrocytes with the *plasmodium* parasite (Yang et al., 2020; Abdurahman et al., 2021), with subsequent improvements reaching precision of 93%, recall of 93% and F1 score of 93%. The initial YOLOv4 network obtained 89.3% ± 0.8% for precision, 83.5% ± 0.8% for recall and 86.0% ± 0.6% for F1 score. After hyperparameter optimisation (using momentum of 0.93, learning rate of 0.0001, and decay of 0.00005 from the beta-A-3 network), the performance improved to 91.6% precision, 89.2% recall, and 90.5% F1 score, bringing the results closer to state-of-the-art performance while observing the cells stain-free and in flow. This optimisation process, while decreasing dataset size, has had a positive effect on the network, potentially removing erroneous examples of each class and optimising feature detection.

Expanding the dataset to include platelets and leukocytes led to a slight decrease in F1 score from 90.5% to 84.2% ± 1.9% when training the YOLOv4 network. Notably, some echinocytes were initially misclassified as erythrocytes and contrariwise, with the confusion matrix showing 43 echinocytes (7.9%) misclassified as erythrocytes and 24 erythrocytes (7.7%) misclassified as echinocytes out of 542 and 311 total cells respectively (Table 3A). This misclassification may be due to their similar sizes and morphology. In the expanded dataset, leukocyte recall was at 85.2% ± 5.2% and precision at 89.9% ± 5.2%, indicating the network was able to retrieve around 85.2% of all leukocytes within the image dataset. Of those, 89.9% were correctly identified as leukocytes (Szegedy et al., 2016). YOLO networks function in two steps: firstly, finding the object, followed by identifying it. Therefore, if an object represents a small portion of the training population, the network would be more likely to be rewarded by identifying the object as another, more represented class of objects. This is why some cells can be misidentified.

While increasing the dataset size is generally beneficial for network performance, the results revealed a more nuanced relationship between dataset complexity and model accuracy. When expanding to the six-class model with over 197,000 images (53,100 echinocytes, 33,200 erythrocytes, 7,100 lymphocytes, 3,300 monocytes, 4,100 neutrophils and 9,600 platelets), the overall network performance decreased to 74.3% ± 0.6% F1 score (Table 4B), compared to the four-class model’s 84.2% ± 1.9% (and even 88.1% ± 1.8% for the retrained 3 class network; Tables 3C,D). The most significant challenge was distinguishing between morphologically similar cell types, as evidenced by the substantial misclassification between echinocytes and erythrocytes, with 5,235 echinocytes misclassified as erythrocytes and 1,457 erythrocytes misclassified as echinocytes. Reclassification of these similar cell types into a single Red Cells category resulted in a performance improvement, with the five-class model achieving an F1 score of 78.9% ± 0.3%, and the simplified three-class model demonstrating comparable results (Red Cells, Leukocytes, Platelets) reaching 87.6% ± 0.5% F1 score (Table 5B). This suggests that optimal network performance depends not only on dataset size and the appropriate grouping of morphologically similar cell types based on specific classification requirements, but also on the balance of the dataset itself, given that red cells outnumber other types by approximately 3 to 4 fold, potentially biasing the network to favour red cell classification at the expense of other cell types (Dawson et al., 2023).

Table 5 presents the results of the optimised three-class model, where all leukocyte subtypes were consolidated into a single Leukocyte class and combined echinocytes and erythrocytes into a unified red cell class. This strategic grouping yielded noteworthy improvements, with the network achieving 84.1% ± 0.9% precision, 92.4% ± 0.2% recall, and 87.6% ± 0.5% F1 score overall. Red cells were detected with the best accuracy (97.9% ± 0.1% precision, 91.2% ± 0.6% recall), while the consolidated Leukocyte class showed substantial improvement over individual leukocyte subtypes (70.3% ± 1.0% precision, 91.9% ± 0.5% recall). The confusion matrix reveals that classification errors occurred primarily at the boundary between cell types, with 2,074 red cells misclassified as leukocytes and 448 leukocytes incorrectly identified as red cells. This pattern likely stems from morphological overlap between larger red cells and smaller leukocytes, particularly challenging without staining agents to provide biochemical differentiation. The size distribution overlap shown in Figure 4 confirms this boundary ambiguity, while class imbalance and imaging limitations further complexify the difficulty in distinguishing cells with borderline characteristics.

This improvement in performance (87.6% F1 score) compared to more granular models suggests that neural networks struggle with subtle morphological distinctions between closely related cell types but excel at broader categorical differentiation. The substantial precision gap between red cells (97.9%) and leukocytes (70.3%), despite high recall across all classes (>90%), highlights a fundamental challenge in unstained blood cell analysis: disambiguating cells with similar morphological profiles. Here, these findings demonstrate that practical AI implementation for routine blood analysis benefits from tailoring classification granularity to specific diagnostic needs rather than maximising taxonomic detail. This principle could inform the development of more effective clinical haematology tools.

Data leakage in object detection refers to the unintentional sharing of information between the training and evaluation datasets, which can result in misleading high-performance metrics. This may occur, for example, if images containing a particular class or even the same objects in slightly different frames appear in both sets. Similarly, artefacts such as consistent illumination patterns or background features may also contribute to leakage. In such cases, the model may learn to recognise specific patterns rather than generalisable features, thereby undermining its ability to perform accurately on truly unseen data (Apicella et al., 2024).

Here, the imaging setup was standardised across all samples, which reduces the likelihood of illumination-related leakage, as identical settings were applied to all images. Placing the prototype within a controlled environment further limited the impact of environmental lighting during data capture. Moreover, the training and test datasets were drawn from different time points within video sequences, making it unlikely that the same cells were present in both sets. While cells from the same donor may appear across datasets, they would not be identical.

However, it is more difficult to completely rule out the influence of background artefacts introduced during individual experiments. Although microfluidic channels were washed and reused up to three times, some single-cell cultures exhibited visible debris. To mitigate this, new channels were introduced after debris was observed, and the data would be used either exclusively for training or testing, thereby reducing the potential for data leakage arising from background artefacts.

The performance metrics (F1-score, precision, and recall) reported in this study are indeed influenced by class imbalance within the dataset, particularly in the six-class model, where leukocyte subtypes such as monocytes and lymphocytes were underrepresented relative to erythrocytes and echinocytes. To mitigate this, we employed stratified 5-fold cross-validation, ensuring that each fold maintained class proportions reflective of the full dataset. Moreover, the performance tended to decline for underrepresented classes (e.g., lymphocytes), particularly in terms of precision, suggesting that the model was more likely to misclassify these cells as morphologically similar types. To address this, class consolidation was explored (e.g., grouping all leukocytes or combining erythrocytes and echinocytes into “Red Cells”), which yielded improved and more balanced performance metrics across classes (Table 5). While class imbalance does impact precision and recall, dataset structuring and cross-validation can reduce its influence. Future research could explore the benefits of training with better balancing between the classes, by subsampling from the full cell pool, for example.

### A comparative analysis with traditional methods

Our approach compares with other networks trained on stained blood smears (Shams et al., 2024). Their blood smear-based artificial neural network, Bio-Net, developed using YOLOv3, achieved an F1 score of 98% for erythrocytes. In comparison, the optimised three-class model (Table 5B) reached 94.5% ± 0.3% for red cells, a close performance considering the approach uses unstained cells in a microfluidic flow chip. Similarly, platelets reached 98% F1 score in the blood smear network, compared to 88.8% ± 1.1% in the three-class system. Potentially losing some platelets passing in the chip outside of the focus plane. For leukocytes, the Bio-Net demonstrates precision and recall of 100% and 98% for lymphocytes, 99% and 98% for neutrophils, and 99% and 98% for monocytes when analysed separately. When examining the specific leukocyte subtype detection (Table 4B), the unstained approach achieved significantly lower performance with F1 scores of 62.1% ± 1.0% for lymphocytes, 67.8% ± 1.5% for monocytes, and 79.3% ± 0.7% for neutrophils; highlighting the particular challenge of distinguishing leukocyte subtypes without staining agents to reveal their distinctive nuclear and cytoplasmic features. The consolidated leukocyte approach achieved 70.3% ± 1.0% precision and 91.9% ± 0.5% recall, with an F1 score of 80.0% ± 0.7%. Overall, the three-class network achieved a macro-average of 84.1% ± 0.9% precision and 92.4% ± 0.2% recall, with an F1 score of 87.6% ± 0.5%.

While these performance differences might translate to substantial variations in clinical applications involving billions of cells, the unstained approach offers distinct advantages in sample preparation simplicity and real-time analysis capability that may outweigh the modest accuracy trade-offs for many point-of-care applications.

Our findings demonstrate that unmodified YOLOv4 architectures with larger training datasets can achieve comparable performance to more complex custom networks for blood cell classification. The previously stated 3 class network metrics, while robust, do not universally exceed those reported by Wu et al.'s SDE-YOLO, which incorporated the Swin Transformer into YOLOv5s to achieve precision rates of 99.5% for white blood cells and 95.3% for red blood cells (Wu et al., 2023). However, their approach struggled with densely populated and blurred blood cell images, and the complex architecture introduced implementation challenges that could limit its practical utility in resource-constrained settings.

Similarly, Benaissa et al. (2024) and Shams et al. (2024) tackled the persistent challenge of overlapping cells through semantic segmentation approaches with impressive training metrics (F1 score of 0.968 and accuracies of 98%–99%, respectively). Both approaches, however, exhibited limitations in real-world application. Benaissa’s work relied on BCCD 2023, which was limited to healthy individuals and contained only 1,328 images, significantly smaller than the dataset. This restriction likely impairs the model’s ability to handle pathological cases or abnormal cell morphologies (Sarker Depto et al., 2023). Meanwhile, Shams’s approach, utilising the BCCD dataset with merely 1,100 test images, showed a performance drop of approximately 10% during validation compared to training (F1 score of 0.909), particularly when analysing densely crowded areas (Supplementary Figure S2).

Interestingly, these results revealed that YOLOv4 outperformed newer YOLO versions in the specific application, contradicting the general assumption that iterative versions would improve performance. Notably, YOLOv4 employs the CSPDarknet53 backbone, a deep and expressive architecture built to extract subtle morphological features in low-contrast or texture-sparse images, such as those of unstained cells. In contrast, YOLOv5 and later versions utilise lighter CSPNet-inspired or Rep-style backbones that prioritise efficiency over depth, potentially limiting their discriminative capacity in fine-grained biomedical tasks. This suggests that architectural advancements in newer YOLO versions may not universally benefit all detection tasks, particularly for unstained blood cell classification, where subtle morphological differences are critical.

Whilst others have limited their investigation to specific cell populations such as CD4^+^ immune cells, this work advances the field by demonstrating comparable performance to established clinical techniques across multiple cell types simultaneously, without requiring staining or complex sample preparation (Dixit et al., 2024). This has promising implications for point-of-care diagnostics in resource-limited settings, where this approach could enable rapid, accurate, and cost-effective blood analysis using relatively modest computational resources.

Looking at the typical output from the artificial neural network, cell size remains consistent with the literature. For platelets, larger platelet size can reach up to 7 μm, which matches up with what is expected in the literature, with most being smaller or equal to 5 µm (Robier, 2020). Likewise, red cells, being mostly between 6 and 8 μm, match up within their expected morphology (Hoffbrand and Lewis, 1989). Lymphocytes remain within 10–15 µm in height and width, which is slightly higher than their typical range of 8–10 µm (Cano and Lopera, 2013). This could be due to the measurements being obtained from the bounding box surrounding the cell instead of the cell itself. If the cell does not have a spherical shape, using the bounding box will slightly overestimate cell size. Here, neutrophils can be found between 12 and 17 µm in width and height, with monocytes being found from 12 to over 22 µm in width and height (Baker et al., 1976). Again, here, the size distribution range appears to agree with typical cell size mostly. However, there are some outliers, these cells could be miss identified by the detector and classified as another cell type. Indeed, a very small portion of monocytes were identified as lymphocytes and large erythrocytes by the detector (Table 4). However well a network is trained, more data will always be required to reduce the occurrence of misclassifications.

The comparison of artificial neural network detection accuracy with flow cytometry and haematology analysers involved conducting tests on unseparated blood samples from donors. Results from all three donors matched up between techniques, and statistical analysis revealed no statistical difference. However, the error bars for the artificial neural network are visibly larger than others. The discrepancy between AI and flow cytometry measurements may be attributed to the underrepresentation of platelets in flow cytometry results, potentially due to platelets being misidentified as cell debris. The deviation in white cell detection likely stems from the enhanced sensitivity of labelled flow cytometry for identifying leukocytes, whilst the AI system may miss some leukocytes due to incomplete sample observation or detection limitations. The apparent variation in leukocytes seen in artificial neural network analysis could be due to sedimentation within the syringe while the sample is pushed through the system. Measurements typically took up to 45 min to be imaged; however, after optimisations, measurements could take up to 25 min; the analysis included comparable ratio results to the longer analysis time. This could be reduced further by using a faster feed rate through the imaging field along with a camera with a higher capture rate, which could reduce this time or analyse more cells within the same period. However, this has not yet been tested.

Additionally, slight variations between diagnostic devices are not uncommon. A review comparing different haematology analysers found variations between analysers from different manufacturers even though they rely on the same core technology (Brue et al., 2015). For example, the largest variation observed was around 20% seen for reticulocyte counts between Cell-Dyn Sapphire and Advia 2120i compared to the median, as well as for monocyte counts, with Advia 2120i being 20% lower than the median. Compared to the artificial neural network, which here has a variation of 6.5% (calculated on comparative tests, Figure 4). Ergo, the variations seen here are not unexpected.

A future consideration could be the integration of programming logic to output the interpretation of the results automatically. While YOLOv4 is the best-performing network so far, it would be worth testing newer YOLO versions past version 7 as well. Having both the population and individual cell data will drastically help determine the prognosis. Additionally, training on cells that have abnormal morphologies will allow the early detection of the initial few cells of a disease or monitoring therapy by assessing cellular response. Another consideration could be to separate a portion of the whole blood and lyse red cells to better analyse the sample, like haematology analysers. Additionally, the analysis happens after the recording rather than concurrently. Further work into using dedicated hardware that can accelerate AI inference, such as the Nvidia Jetson nano, to enable concurrent analysis to recording, giving near instantaneous results.

## Conclusion

This study has successfully employed YOLO v4 to detect and discriminate blood cell sub-populations, beginning with a binary classification of erythrocytes and echinocytes. While encountering a slight decrease in accuracy during the expansion to include platelets and leukocytes, with occasional misclassifications, subsequent iterations and model modifications showcased remarkable accuracy in distinguishing various cell types, particularly achieving a promising 99% accuracy in the final evaluation for echinocytes. The combination of artificial neural networks and microscopy demonstrated its effectiveness in blood cell discrimination and classification and illuminated a promising path for breakthroughs in medical diagnostics. This success lays the groundwork for continued advancements through ongoing model refinement and exploring innovative approaches.

## Data Availability

The raw data supporting the conclusions of this article will be made available by the authors, without undue reservation.
