# Supplementary material for: Stain-free artificial intelligence-assisted light microscopy for the identification of blood cells in microfluidic flow
Source: Front Bioinform. 2025 Aug 14;5:1628724. doi: 10.3389/fbinf.2025.1628724 (PMC12391159; doi:10.3389/fbinf.2025.1628724)
Supplement: Supplementary file 1 [file DataSheet1.docx]

Supplementary data to Stain-free artificial intelligence-assisted light microscopy for the identification of blood cells in microfluidic flow.

Alexander Hunt^1^, Holger Schulze^1^, Kay Samuel^2^, Robert B. Fisher^3^, Till T. Bachmann^1^

1. Centre for Inflammation Research, Institute for Regeneration and Repair, The University of Edinburgh
2. Tissues, Cells & Advanced Therapeutics, Scottish National Blood Transfusion Service, NHS National Services Scotland, Jack Copland Centre, 52 Research Avenue North
3. School of Informatics, The University of Edinburgh

Correspondence author: Prof. Till Bachmann, till.bachmann@ed.ac.uk

Keywords: Artificial Neural Network, morphological analysis, YOLO v4, blood analysis

Figures:

[Figure S1: example of annotated tiles with erythrocytes labelled in pink and echinocytes in green. 2](#_Toc201658598)

[Figure S2: example of a crowded field of view with cells overlapping others. Depicted here are a mix of echinocytes and erythrocytes. 2](#_Toc201658599)

[Figure S3: YOLO v4 finds and correctly identifies most echinocytes and erythrocytes that are clear from the edges. 3](#_Toc201658600)

[Figure S4: Comparative plot of different iterations of YOLOv4 on the binary dataset. Each version has precision, recall, accuracy, and F1-score for each network outlined in Table S1. Plotted in percentage total of 1. 5](#_Toc201658601)

[Figure S5: Comparison images of leukocytes labelled using bounding boxes defined in the ground truth (right) and the predicted boxes from the YOLO v4 neural network trained on six cell types. 6](#_Toc201658602)

[Figure S6: YOLO versions 4 to 7 trained on the three-class dataset. Each network was trained with five-fold validation and plotted the precision, recall, F1 score, and accuracy from 5-fold validation training with standard deviation as error bars n=5. Statistical significance is indicated by asterisks: * p < 0.05, ** p < 0.01, *** p < 0.001, **** p < 0.0001 7](#_Toc201658603)

Tables:

[Table S1: table describing modulation of YOLO network hyperparameters (decay, learning rate and momentum). 4](#_Toc201658604)

[Table S2: p-value correlation table 6](#_Toc201658605)


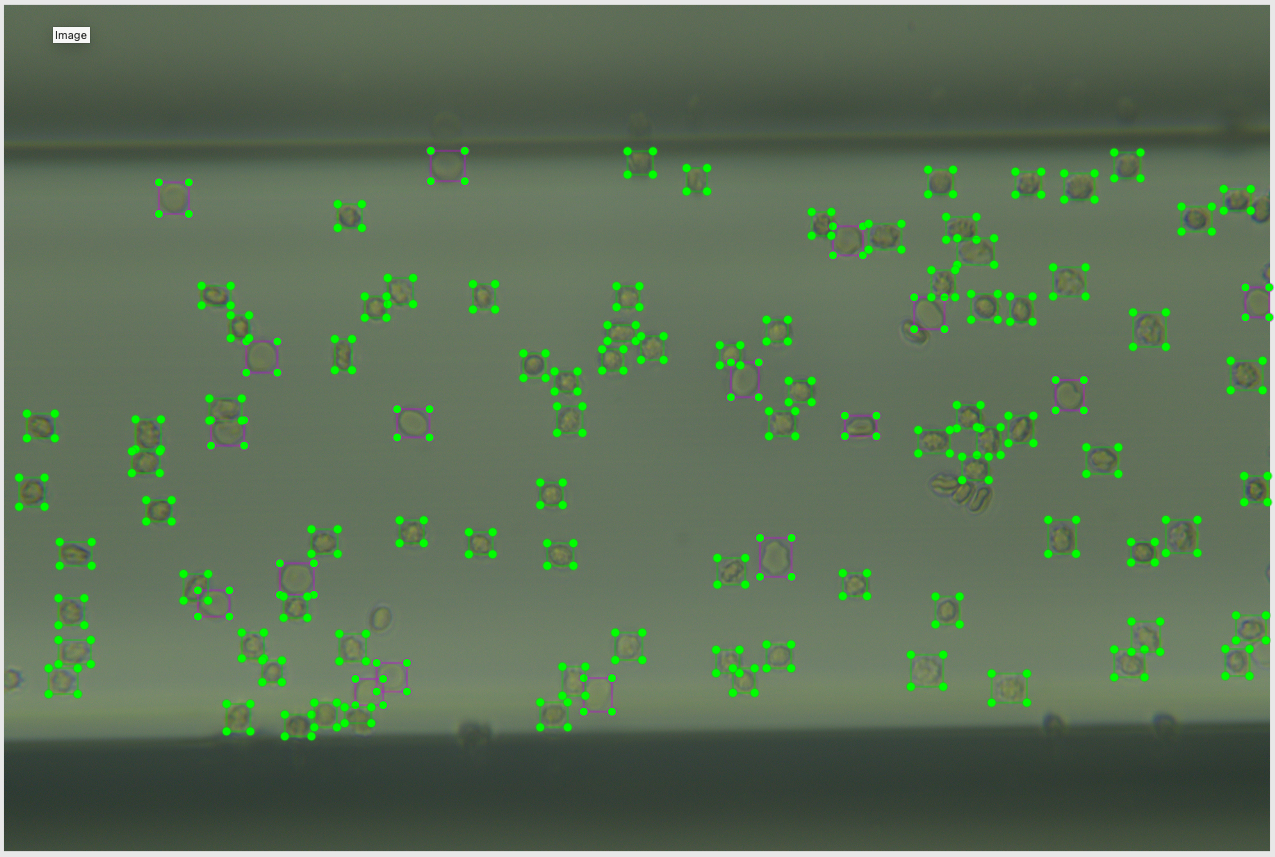


Figure S1: example of annotated tiles with erythrocytes labelled in pink and echinocytes in green.


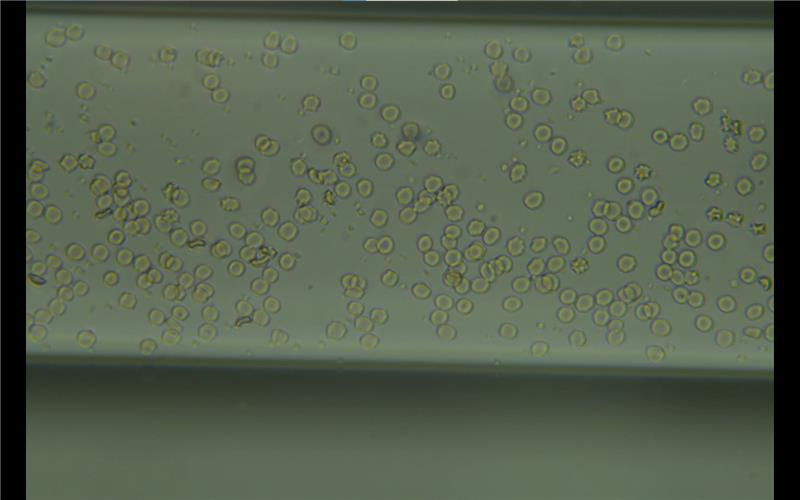


Figure S2: example of a crowded field of view with cells overlapping others. Depicted here are a mix of echinocytes and erythrocytes.


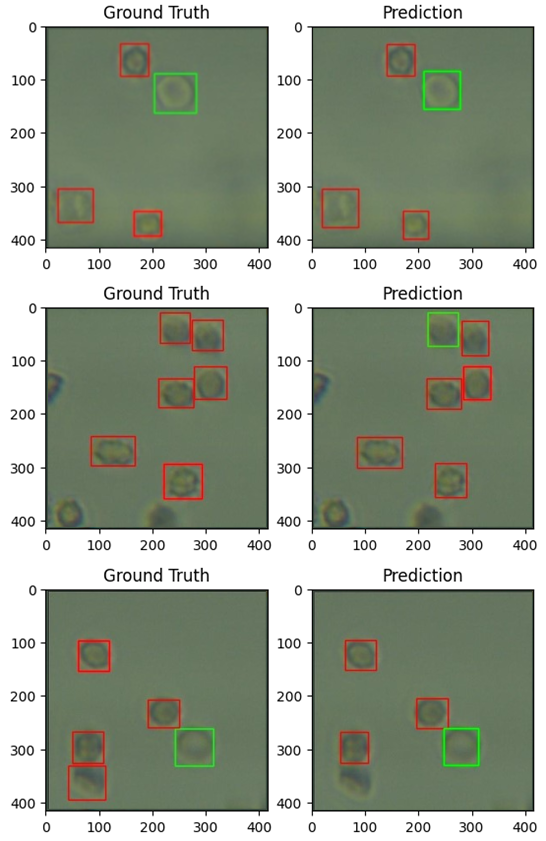


Figure S3: YOLO v4 finds and correctly identifies most echinocytes and erythrocytes that are clear from the edges.

The red bounding box labels echinocytes, and the green labels erythrocytes. Images on the left are labelled as per the ground truth bounding boxes and with the predicted boxes on the right The second image set has an instance of misclassification of an echinocyte as an erythrocyte. The last image set misses an erythrocyte. This model was trained using over one thousand images of cells in the training set. The dimensions of the images are 416x416 pixels, or 57.37x57.37 µm. The cells were imaged at 40x zoom

Table S1: table describing modulation of YOLO network hyperparameters (decay, learning rate and momentum).

Each cell describes the momentum / decay / learning rate for a specific network. Each iteration is named using the reference laid out in the tables, e.g. for momentum / decay /learning rate of 0.94 / 0.005 / 0.001 respectively the network name is gamma – B – 1. Cells coloured in green led to successfully trained networks. Cells coloured in yellow resulted in models which could not complete training successfully.

| Decay - 0.005 - 1 |  |  |  |  |  |  |
| --- | --- | --- | --- | --- | --- | --- |
| Momentum \ learning rate | 0.00001 - alpha | 0.0001 - beta | 0.001 (default) - gamma | 0.01 - delta | 0.1 - epsilon |  |
| 0.93 - A | 0.93 / 0.005 / 0.00001 | 0.93 / 0.005 / 0.0001 | 0.93 / 0.005 / 0.001 | 0.93 / 0.005 / 0.01 | 0.93 / 0.005 / 0.1 |  |
| 0.94 - B | 0.94 / 0.005 / 0.00001 | 0.94 / 0.005 / 0.0001 | 0.94 / 0.005 / 0.001 | 0.94 / 0.005 / 0.01 | 0.94 / 0.005 / 0.1 |  |
| 0.95 (default) - C | 0.95 / 0.005 / 0.00001 | 0.95 / 0.005 / 0.0001 | 0.95 / 0.005 / 0.001 | 0.95 / 0.005 / 0.01 | 0.95 / 0.005 / 0.1 |  |
| 0.96 - D | 0.96 / 0.005 / 0.00001 | 0.96 / 0.005 / 0.0001 | 0.96 / 0.005 / 0.001 | 0.96 / 0.005 / 0.01 | 0.96 / 0.005 / 0.1 |  |
| 0.97 - E | 0.97 / 0.005 / 0.00001 | 0.97 / 0.005 / 0.0001 | 0.97 / 0.005 / 0.001 | 0.97 / 0.005 / 0.01 | 0.97 / 0.005 / 0.1 |  |
| Decay - 0.0005 - 2 |  |  |  |  |  |  |
| Momentum \ learning rate | 0.00001 - alpha | 0.0001 - beta | 0.001 (default) - gamma | 0.01 - delta | 0.1 - epsilon |  |
| 0.93 - A | 0.93 / 0.0005 / 0.00001 | 0.93 / 0.0005 / 0.0001 | 0.93 / 0.0005 / 0.001 | 0.93 / 0.0005 / 0.01 | 0.93 / 0.0005 / 0.1 |  |
| 0.94 - B | 0.94 / 0.0005 / 0.00001 | 0.94 / 0.0005 / 0.0001 | 0.94 / 0.0005 / 0.001 | 0.94 / 0.0005 / 0.01 | 0.94 / 0.0005 / 0.1 |  |
| 0.95 (default) - C | 0.95 / 0.0005 / 0.00001 | 0.95 / 0.0005 / 0.0001 | 0.95 / 0.0005 / 0.001 | 0.95 / 0.0005 / 0.01 | 0.95 / 0.0005 / 0.1 |  |
| 0.96 - D | 0.96 / 0.0005 / 0.00001 | 0.96 / 0.0005 / 0.0001 | 0.96 / 0.0005 / 0.001 | 0.96 / 0.0005 / 0.01 | 0.96 / 0.0005 / 0.1 |  |
| 0.97 - E | 0.97 / 0.0005 / 0.00001 | 0.97 / 0.0005 / 0.0001 | 0.97 / 0.0005 / 0.001 | 0.97 / 0.0005 / 0.01 | 0.97 / 0.0005 / 0.1 |  |
| Decay - 0.00005 - 3 |  |  |  |  |  |  |
| Momentum \ learning rate | 0.00001 - alpha | 0.0001 - beta | 0.001 (default) - gamma | 0.01 - delta |  |  |
| 0.93 - A | 0.93 / 0.00005 / 0.00001 | 0.93 / 0.00005 / 0.0001 | 0.93 / 0.00005 / 0.001 | 0.93 / 0.00005 / 0.01 |  |  |
| 0.94 - B | 0.94 / 0.00005 / 0.00001 | 0.94 / 0.00005 / 0.0001 | 0.94 / 0.00005 / 0.001 | 0.94 / 0.00005 / 0.01 |  |  |
| 0.95 (default) - C | 0.95 / 0.00005 / 0.00001 | 0.95 / 0.00005 / 0.0001 | 0.95 / 0.00005 / 0.001 | 0.95 / 0.00005 / 0.01 |  |  |
| 0.96 - D | 0.96 / 0.00005 / 0.00001 | 0.96 / 0.00005 / 0.0001 | 0.96 / 0.00005 / 0.001 | 0.96 / 0.00005 / 0.01 |  |  |
| 0.97 - E | 0.97 / 0.00005 / 0.00001 | 0.97 / 0.00005 / 0.0001 | 0.97 / 0.00005 / 0.001 | 0.97 / 0.00005 / 0.01 |  |  |


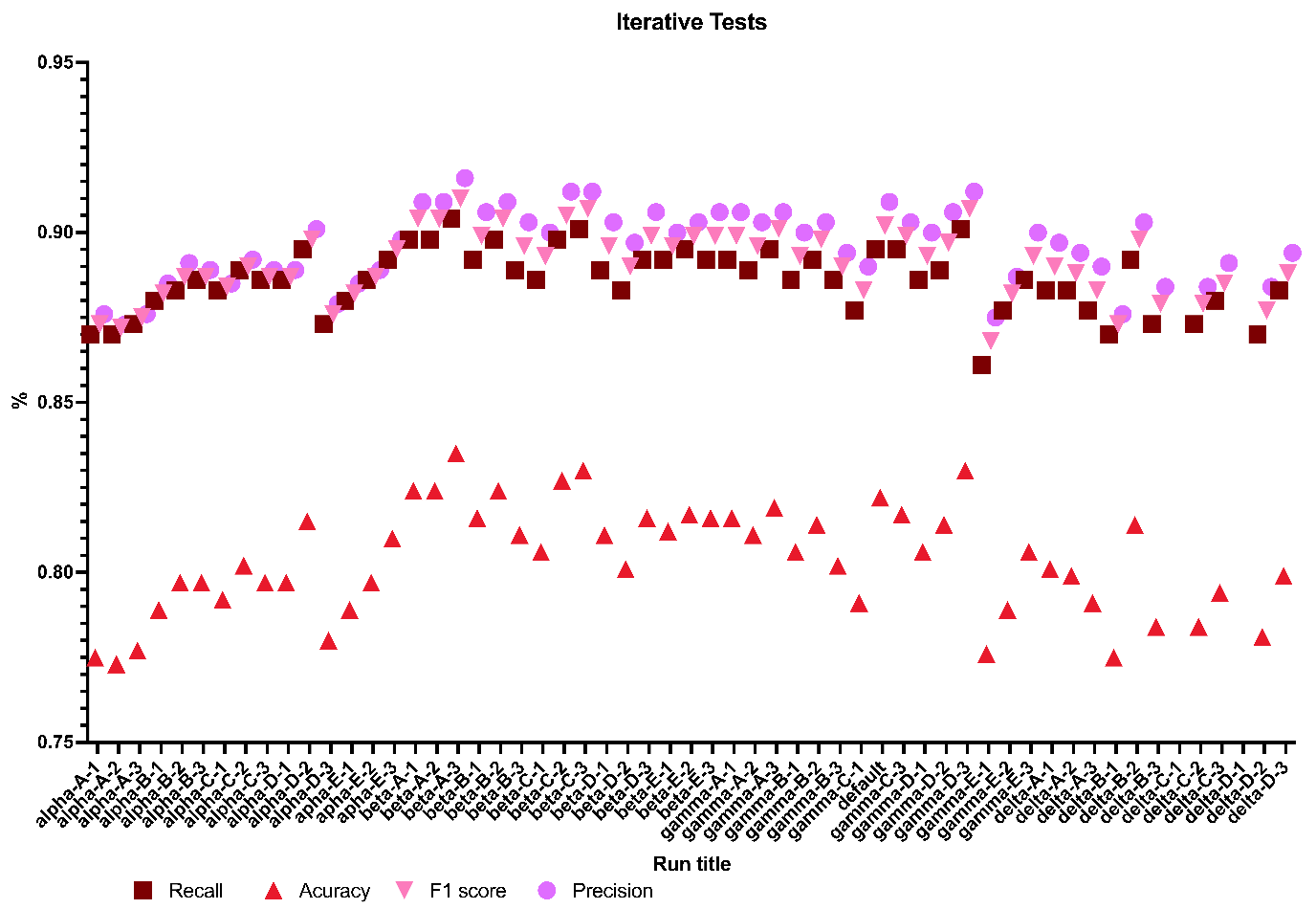


Figure S4: Comparative plot of different iterations of YOLOv4 on the binary dataset. Each version has precision, recall, accuracy, and F1-score for each network outlined in Table S1. Plotted in percentage total of 1.


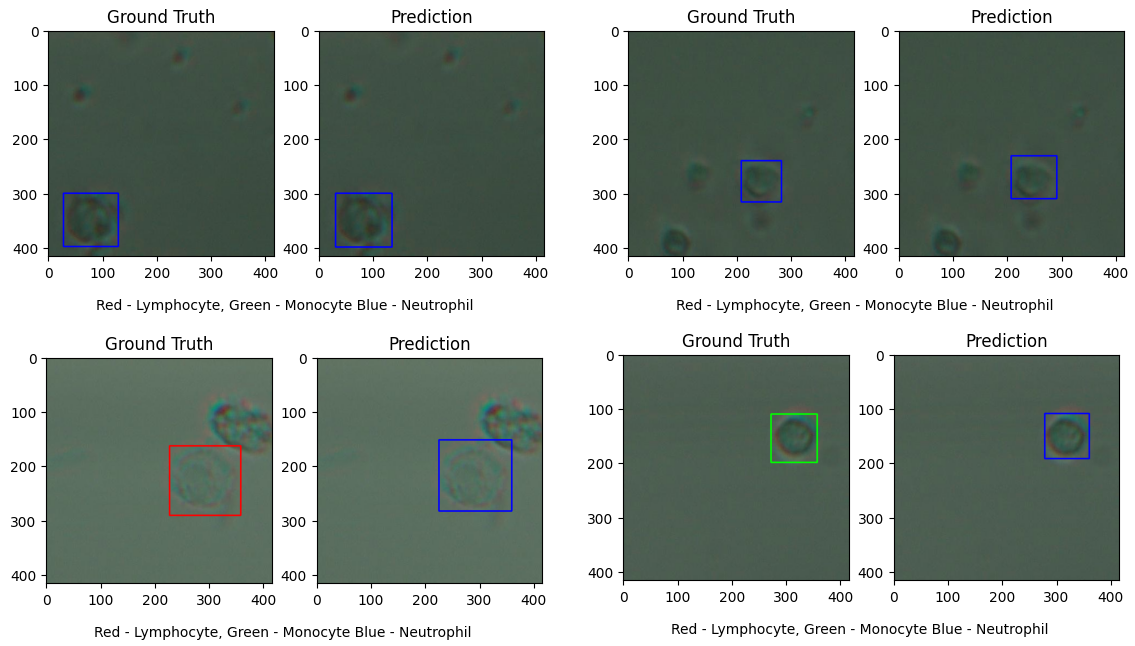


B

A

D

C

Figure S5: Comparison images of leukocytes labelled using bounding boxes defined in the ground truth (right) and the predicted boxes from the YOLO v4 neural network trained on six cell types.

The first two examples are correct identifications of neutrophils (labelled in blue here A, and B). The third image set represents a lymphocyte (red) identified as a neutrophil (C). Finally, the fourth set represents a monocyte (green) identified as a neutrophil (D) The dimensions of the images are 416x416 pixels or 57.37x57.37 µm. The cells were imaged at 40x zoom.

Table S2: p-value correlation table

Correlation table of randomised blood cell concentration samples comparing labelled flow cytometry and a custom-trained YOLO v4 object detector major sub-population ratio (trained to recognise echinocytes, erythrocytes, lymphocytes, monocytes, neutrophils, and platelets). Erythrocyte rations are observed to be mostly consistent along the correlation line. Platelets are seen to be overrepresented in the AI device, while white cells tend to be more detected by flow.

|  | | AI | | |
| --- | --- | --- | --- | --- |
|  |  | Erythrocytes | Platelets | Leukocytes |
| Flow Cytometry | Erythrocytes | 0.0000865 | 0.0002 | 0.0351 |
|  | Platelets | 0.0174 | 0.0022 | 0.0568 |
|  | Leukocytes | 0.2698 | 0.071 | 0.0297 |

Figure S6: YOLO versions 4 to 7 trained on the three-class dataset. Each network was trained with five-fold validation and plotted the precision, recall, F1 score, and accuracy from 5-fold validation training with standard deviation as error bars n=5. Statistical significance is indicated by asterisks: * p < 0.05, ** p < 0.01, *** p < 0.001, **** p < 0.0001

## Flow Cytometry Gating Strategy
